# Urologists and social media: pitfalls and perils and what to think about before hitting *‘send’*

**DOI:** 10.1111/bju.16636

**Published:** 2025-01-13

**Authors:** Helen W Cui, John Reynard

**Affiliations:** ^1^ Department of Urology Roswell Park Comprehensive Cancer Center Buffalo New York USA; ^2^ Department of Urology Churchill Hospital Oxford UK

**Keywords:** fitness to practise, regulation, social media, twitter, whatsapp

## Abstract

**Objective:**

To summarise current guidelines from professional bodies relevant to urologists on social media, and to discusses a range of risks associated with social media use. These include the risk of a fitness to practise investigation, breaking the law, loss of employment, and personal risk in the form of harassment and doxxing.

**Methods:**

Review of guidelines and recommendations published by professional bodies revelant to urologists and review of relevant case examples in the medical profession and other relevant professions.

**Results:**

This article finds whereas most doctors will be aware of the risks of posting on public social media platforms, the recent case studies in medical and other contexts have highlighted the risks of disciplinary action from regulators and even criminal investigation in the use of private social media.

**Conclusion:**

Although the majority of urologists are unlikely to violate ethical and good medical practice principles in their use of social media, this article serves as a reminder of the potential consequences based on real‐life case examples.

**Abbreviations**
AHPRA: Australian Health Practitioner Regulation Agency
GMC: General Medical Council
NDA: non‐disclosure agreement
PIDA: Public Interest Disclosure Act

## Introduction

The summer 2024 riots in England and Wales led to significant prison sentences handed down to individuals who had posted material online intended to stir up racial hatred. We were reminded by the Prime Minister, Sir Keir Starmer, that social media ‘is not a law‐free zone’ [1].

Whilst it is hard to imagine any doctor being involved in criminal activity of this nature, this statement should serve as a stark warning that social media platforms are a potential source of regulatory risk for the medical profession.

The purpose of this review is to bring to the attention of the wider urological community the potential dangers of social media and to summarise current guidelines that govern the use of social media platforms. This review will highlight recent case examples where a breach of these guidelines led to regulatory, or criminal proceedings against doctors in the hope that urologists can avoid these potential pitfalls in their use of social media.

## Social Media: Definitions, Benefits and Ignorance of Guidelines Governing Its Use

The General Medical Council (GMC) in the United Kingdom defines social media as including ‘the use of private messaging, websites and applications that enable users to create and share content, or to participate in social networking’ [2].

Social media allows the sharing of information, discussion of ideas, the democratic dissemination of academic data, promotion of a personal profile, networking and marketing. Urologists, in particular, have embraced 𝕏 (formerly Twitter) as a platform, with nearly 40% of academic urologists in the United States having an 𝕏 profile [3]. Activity on 𝕏 during urological conferences has been shown to be triple that of other major non‐urological surgical conferences [4]. Although the majority of the global top 100 𝕏 urology influencers are United States‐based (64%), the next highest proportion are UK urologists (10%) and, overall, these influencers have an average of over 5000 followers [5]. The potential impact of this global platform as a means of reaching peers and the public is significant [6].

As with any tool, an awareness of professional guidelines and the potential personal, professional and legal implications of social media use is important. An American Urological Association (AUA) survey suggests that, whilst nearly three‐quarters of practising urologists had a social media account, 53% were not familiar with professional guidelines on social media use [7].

Urologists need to be aware of the remit that both regulatory bodies (e.g., the GMC in the United Kingdom) and the police have in investigating any social media content which may potentially breach professional guidelines or civil or criminal law. The concept that a doctor, through a breach of guidelines, may be at risk of compromising their fitness to practise is not abstract. A search of all published cases investigated by the UK Medical Practitioners Tribunal Service in the last 12 months (excluding those linked to a criminal conviction) revealed nine cases that included the use of social media. Of these, four cases were related to publicly voicing misinformation related to the COVID‐19 pandemic and vaccination, three included the use of social media during relationships with patients, one included the posting of offensive messages on Twitter, and one was linked to inaccurate claims about the efficacy of an i.v. infusion business being advertised on social media. The outcomes of the Medical Practitioners Tribunal Service decision for these cases resulted in one warning, three suspensions and five erasures from the UK medical register. In the United States, a survey of the directors of medical licensing boards found that 92% had at least one case of online professionalism violations ever reported to their board, of which the most common were inappropriate patient communication, use of the internet for inappropriate practice, and online misrepresentation of credentials [8].

## Professional Standards Guidance on Social Media Use for Doctors

Table 1 outlines the recommendations and statements published by the GMC [2], the *BJUI* [9], the British Medical Association (BMA) [10], the European Association of Urology (EAU) [11] and the AUA [12]. The regional Canadian colleges, the Canadian Medical Protective Society and the Australian Medical Board also provide guidance on social media use [13, 14, 15, 16, 17]. Similar themes emerge across the different guidelines, which generally follow the ethical principles that all doctors are familiar with and over which there is no debate.

**Table 1:** Professional standards guidelines for social media use for doctors.

Guideline	GMC [2]	*BJUI* [9]	BMA [10]	EAU [11]	AUA [12]
Last updated	2024	2014	2018	2018	2024
Behaving professionally	You must make sure your conduct justifies patients' trust in you and the public's trust in your profession.	Align your digital profile and behaviour with standards of your profession.	Take a cautious approach to anything that you think could affect your professional standing.		Be professional.
Identifying yourself	If you identify yourself as a doctor in publicly accessible social media you should also identify yourself by name.	If you are posting as a doctor you should identify yourself as such.	You are still a doctor or medical student on social media even if you don't identify yourself as such or post medical matters.	Stating your name, who you are and your profession is key in establishing a professional digital identity.	If you identify your affiliation with the AUA, your social media activities must be consistent with the AUA's professional Code of Ethics.
Giving expertise	Give appropriate context to your content to help the audience verify your claims and expertise. Be honest and trustworthy in your communications.	Always strive for accuracy and truth.	Avoid providing personalised health advice via social media to members of the public.	Avoid providing medical advice.	
Being courteous	You must not use social media to abuse, discriminate against, bully, harass or deliberately target any individual or group.		It is illegal to threaten or harass someone online.		Be courteous. Refrain from using threatening or discriminatory remarks, personal insults or obscenities.
Maintaining boundaries	Maintain a professional boundary between you and your patient.	It is common for patients to be interested in physicians' social profiles.	Protect your privacy online. How secure or private you want to make your social media activity is up to you.	Maintain limits between your social media presence and patients to ensure professional decorum	
Maintaining confidentiality	Do not share identifiable information about patients even on private sites or forums.	Protect patient privacy and confidentiality.	Think carefully about the information you include and whether you will need to get consent from patients.	Never underme patient privacy and confidentiality.	Never post or disclose protected health information including information that may allude to or actually identify a patient whatever the format may be.
	The GMC has two additional guidelines for use of patient information for educational purposes.		Follow GMC guidance and get patient consent to share images of patients or cases.		
Respect for others	Treat colleagues fairly and with respect in all situations and all forms of interaction and communication.	Being respectful and not posting in anger is recommended.			
Copyright and defamation	Online postings in a personal or professional capacity are subject to the same laws of copyright and defamation as verbal or written communications.	State your views are your own if your institutions are identifiable.	You have rights to free speech but they are not absolute. Avoid adverse comments about individuals or organisations which could be perceived to be gratuitous or unsubstantiated.	Be courteous and avoid defamatory comments	Exercise discretion. Be mindful of copyright and plagiarism laws when publishing someone else's work.
Conflicts of interest	Be open about any conflict of interest including financial and commercial interests in healthcare organisations or pharmaceutical and biomedical companies.	Social media can have a strong influence on patients. Avoid impropriety and disclose potential conflicts of interest.	If you are posting as a doctor, your posts are likely to be taken on trust. Follow GMC guidance on conflicts of interest.	Be aware of how advertisement and self‐promotion is perceived by others.	
Private communication	Private communications may become public.		Whatever privacy options you choose, your privacy can never be guaranteed, even in closed groups or forums.		
				Use instant messaging software with care as not all are HIPAA compliant.	
	The GMC has a legal duty to investigate any concern raised to them that reaches their fitness to practise threshold.	It should be assumed all platforms lack privacy.	A good rule is never share or reveal anything on social media that you would not be happy to see printed in a newspaper.		
Permanence		Content will exist forever and be available to everyone.		Assume permanence of content.	Be thoughtful. Remember, what you publish will be public for a long time.

AUA, American Urological Association; BMA, British Medical Association; EAU, European Association of Urology; GMC, General Medical Council.

The GMC professional standard guideline ‘Using social media as a medical professional’ came into effect in January 2024 [2]. Although this is a set of ‘recommendations’ for social media use, these highlight that doctors must take note of the fundamental underlying principle of ‘Trust and Professionalism’ as outlined in the GMC's ‘Good Medical Practice’. This states that *‘you must make sure your conduct justifies patients' trust in you and the public's trust in your profession’*. The emphasis on public trust in the profession, combined with the statutory obligation that *‘the GMC has a legal duty to investigate any concern raised to them that reach their fitness to practise threshold’*, means that social media use which could be perceived to undermine trust in an individual doctor or in the medical profession is considered a fitness to practise issue. This is regardless of whether the content is related in any way to the job of being a doctor, i.e., the guidelines apply to both professional *and* personal use of social media.

The GMC has previously stated that *‘the GMC has no interest in doctors' use of social media in their personal lives – Tweets, blogs, Facebook pages etc. But doctors mustn't undermine public trust in the profession. Usually this means breaking the law, even where the conviction is unrelated to their professional life’* [18]. However, what has become increasingly clear is that doctors' use of *private* social media may also be subject to regulatory oversight and be deemed a fitness to practise issue even when it does not meet the threshold of a crime.

## Risks of Private Social Media Use

While urologists using social media may be aware of the potential risks associated with having a public profile (including the impact on their privacy and reputation), not all doctors are aware of the risks that their private communications may pose. A recent number of high‐profile cases of professionals being investigated for private WhatsApp group conversations has highlighted this risk. Professionals implicated in these cases have included doctors, police officers and military personnel. Naïvely, all of the professionals involved assumed that their messages were private or in a private forum. No messages exchanged electronically are truly private. Any content that is posted or shared has the potential to remain forever in the public domain, from which it can be accessed.

Whilst messaging services such as WhatsApp and Apple iMessage have end‐to‐end encryption, unlike text messages, the police can still access these messages if one party submits or is compelled to submit their phone to be examined for evidence. Secrecy in messaging apps such as Signal, where messages are set to disappear, can be easily bypassed by screenshotting the message, as demonstrated in the 2023 fraud case of cryptocurrency exchange founder Sam Bankman‐Fried [19]. Screenshots of his auto‐delete Signal messages sent between colleagues were used as evidence in court. One can safely assume that if someone wishes to preserve one's messages, a way can be found to do so.

## Evidence for the Danger of Private Social Media Use: the ‘WhatsApp Group’ Case

As discussed above, the concept that the use of social media can potentially compromise a doctor's fitness to practise is no longer abstract. In a 2021 case, a group of 10 doctors, referred to by the GMC as ‘the WhatsApp Doctors’, swapped what one newspaper report described as ‘offensive’ messages and pictures on a ‘private’ WhatsApp group between February 2014 and October 2016 [20]. The Professional Standards Authority described the content of the messages as ‘*offensive, racist, discriminatory, and disrespectful towards women, disabled people and people who are LGBTQ*' [21]. As well as written messages, one doctor shared a category A pornographic image and others shared extreme pornographic images’ [22].

Having faced a police investigation (and a potential criminal charge) the group then faced a GMC investigation (a classic case of multiple jeopardy). The GMC argued that each doctor had failed to remove themselves from the group or report the contents, that in so doing they brought the profession into disrepute and that they had impaired their fitness to practise. The doctors argued in the High Court that they should remain anonymous. This argument was rejected by Mrs Justice Eady, who concluded that ‘private communications are not exempt from consideration in professional disciplinary proceedings’ and their names were published in the Daily Mail [20, 22].

Offensive content on public and private forums can be liable to criminal prosecution in the United Kingdom under the Malicious Communications Act of 1988 and the Communications Act of 2003. Although most high‐profile convictions under this Act have related to public postings across social media platforms, including Facebook, Instagram, YouTube and Twitter, recent convictions of police officers for offensive content in WhatsApp messaging groups demonstrate the scope of these Acts to include private communications. Two police officers were convicted for offensive messages found during the course of the Sarah Everard murder investigation [23], and another four ex‐police officers were convicted and given suspended prison sentences for racist content in a WhatsApp messaging group formed after retirement when one member of the group reported the messages to journalists [24]. Specifically, the wording of the Communications Act 2003, section 127 states that a person is guilty of an offence if they:

- send by means of a public electronic communications network a message or other matter that is grossly offensive or of an indecent, obscene or menacing character; or
- cause any such message or matter to be so sent [25].

A ‘public electronic communications network’ is defined as ‘an electronic communications network provided wholly or mainly for the purpose of making electronic communications services available to members of the public’ and therefore includes any service that members of the public can use in order to send electronic signals. This sets a precedent. We might think our personal communications are private. They are not.

In addition to professional regulatory codes and the criminal law, most healthcare employers will have a corporate policy on social media use and breaching this could put one's job at risk. According to an investigation by *The Times* newspaper, over 1200 NHS staff were sanctioned by their employer for social media use between 2013 and 2018, including breaches of patient confidentiality and derogatory messages about colleagues [26].

Other professional organisations have taken a strict view on public and private social media use. In the British Army's view, there are no boundaries between professional and personal life. The Army's policy on social media *‘Applies whether on duty, off duty or on leave … the way you act online should not be different to how you act face to face’* [27]. The Royal Air Force reminds its personnel to *‘make sure your posts do not contain the following … Offensive or threatening language (including swearwords)’* [28].

The balance between maintaining the public's trust in the profession, and the right to a private life and freedom of expression has been, and no doubt will continue to be, argued in both the courtroom and the court of opinion [29]. In a 2011 poll of whether the GMC should be allowed to regulate doctors' private lives, 94% of the 1167 respondents (doctors, patients and the public) thought that the GMC should not regulate doctors' lives outside medicine [30]. A paper investigating the social media profiles of surgical trainees was retracted from the *Journal of Vascular Surgery* after it received criticism for being unethical and unfairly targeting and judging the trainees for their social media content [31].

Doctors do have a right to a private life but must be aware of the legal obligation of the GMC to investigate any allegation related to fitness to practise, which can include behaviour outside of the workplace.

## Risks of Public Social Media Use

The risks of public social media use are more self‐evident. These include risk of breaching patient confidentiality, risk of spreading misinformation, risk of conflict of interest, risk of libel, as well as personal risk to the doctor [32]. Doctors will usually be familiar with the principles of good practice relating to not breaching patient confidentiality on social media and conflict of interest when engaging in activities which may be classed as ‘advertisement’. However, recent events have also highlighted the reputational and personal risks of doctors' public opinions expressed on social media.

### Personal Opinions in a Public Forum

In the past year, social media posts by doctors related to the Israel‐Gaza war have led to a number of reports of doctors being reported to regulators and being fired from their jobs in the United Kingdom, United States and Australia [33]. Information shared by the GMC with *Pulse* news showed there were 377 complaints made to the GMC between October 2023 and March 2024 for antisemitism and Islamophobia, up from just 11 complaints in 2022 [34]. Fourteen of the 377 complaints were under investigation. The Medical Protection Society stated that these referrals included social media posts made on public platforms as well as private WhatsApp messages.

The Medical Defence Union has advised that doctors only post information on private messaging groups if they would be happy for it to be made public [34]. A GMC spokesperson has said *‘Like all citizens, doctors are entitled to personal beliefs, and there is nothing preventing doctors from exercising their right to speak about or campaign on issues, but this must not affect their relationship with patients, or the treatment they provide or arrange. Our guidance is clear that discrimination is not compatible with the responsibilities and duties of a doctor’* [34]. In addition to risk of investigation by the professional regulator and risk of job termination, there is the added personal risk to the doctor making the post, including being the subject of online trolling and doxxing (the gathering and publication of personal information such as addresses and phone numbers by hostile parties to try to intimidate or direct violence at someone).

### Harassment and Doxxing

Personal risks of social media communication include being reported to the regulator as a form of ‘weaponising’ the complaint process, being personally harassed, and doxxing of personal information. For those who wish to bring about negative effects for the original poster, doxxing is the publishing of private and personal identifying information usually with malicious intent. For example, in an article describing the experience of healthcare professionals reported to the Australian Health Practitioner Regulation Agency (AHPRA) for social media posts related to the Israel‐Gaza war, it was reported that closed Facebook groups (consisting mainly of doctors) were being targeted by outsiders joining to capture screenshots of messages and reporting the doctors with malintent to the AHPRA [35].

### Misinformation and Defamation

The need for accuracy and truth, to be trustworthy, and to avoid providing medical advice via social media are noted across the guidelines from the professional bodies (Table 1). The GMC guideline states that *‘It is important that content includes appropriate context, so that people who access what you say about health and healthcare have information that supports their understanding and helps them verify your claims and expertise. If you're commenting on health or healthcare you should usually say who you are’* [2]. A UK surgeon was initially suspended for 6 months after GMC investigation for spreading COVID misinformation on YouTube and Twitter, and then was eventually struck off for repeating his behaviour [36].

Another form of misinformation is relevant to the posting of academic research and medical evidence in online content. Social media has been well utilised by urologists to publicise new research that has been published in peer‐reviewed journals and generate interesting online debate. Social media activity is also seen as advantageous for career enhancement and academic promotion [37]. However, when the medical evidence is published without peer review and the ability to scrutinise the data, this could also have a negative impact on the public's trust in the profession and on the urological community when trying to assess the best evidence‐based medicine for their patients [38]. Using social media as a platform can amplify certain viewpoints and diminish others, amplifying certain biases depending the number of followers of the user. Issues such as conflicts of interest and financial disclosures are also not stipulated in social media posts, and this can undermine professional and public trust in medical research [39].

Doctors risk being accused of libel (the civil offence of a published statement that is damaging to a person's reputation) for posting defamatory content on social media, but are also at risk themselves of being defamed on social media via online reviews. In a case of a doctor being accused of libel, TV personality Dr Christian Jessen was sued for libel after posting a tweet in 2020 defaming Northern Ireland First Minister Arlene Foster. The High Court found in her favour and Dr Jessen was ordered to pay substantial damages and legal costs. Although complaints were made to the GMC, Dr Jessen was not investigated by the GMC, who deemed his actions to be free speech [40].

### Online Reviews by Patients

In the opposing scenario, doctors also face the problem of themselves being defamed, either by other medical professionals and the public, and their own patients through patient online reviews. Patients may post reviews and comments across a number of social media platforms including Facebook, 𝕏 and Reddit, and more commonly on doctor review websites such as WebMD in the United States and Doctify in the United Kingdom.

Whereas most reviews are positive, doctors face the challenge of dealing with negative and sometimes fake reviews, as websites often will not ask for verification that the review reflects a real patient visit [41]. It can be very difficult to respond directly to an online review without the risk of violating patient confidentiality [42].

Doctors are advised to resolve any complaints with patients directly and can also contact the website to ask for the review to be removed if they can prove it is not real. As a last resort, doctors can take legal action for defamation, but the patient will invoke the defence of a right of freedom of speech and any legal claim will always be a costly, time‐consuming and a distressing process for both parties. A case involving the Cleveland Clinic started as a series of reviews by a patient of his urologist, and escalated to lawsuits being filed by several patients against the Cleveland Clinic for malpractice and fraud. The urologist counter‐filed against his patient who was found guilty of felony charges related to harassment and stalking [43].

To counter these issues, doctors can pay for an Online Reputation Management service consisting of software which monitors reviews and may allow reviews to be removed [44]. Asking the patient to sign a non‐disclosure agreement (NDA) which waives their right to post unauthorised online reviews is another mechanism that has been used in the United States [42]. However, there have also been cases of egregious action by doctors to protect their online profile. A Manhattan‐based orthopaedic surgeon violated New York business laws by supressing bad reviews (in some cases offering patients a refund to remove the review) and creating fake positive reviews across several websites [45]. In another case, a Seattle‐based plastic surgeon violated Washington State consumer protection law when he illegally restricted patients from posting negative reviews, directed his staff to post fake positive reviews, and forced patients to sign NDA contracts, resulting in a $5 million fine [46].

### Whistleblowing on Social Media

Whilst it is a professional obligation to raise concerns (whistleblowing), doing so, often anonymously, on social media risks violating employment contracts, but also risks breach of confidentiality, libel and breach of good medical practice. Whistleblowers face the risk of suspension, referral to their professional regulator and legal action over breach of patient confidentiality if they turn over case information to the media [47]. Employees are entitled to legal protection to not be treated unfairly or lose one's job under the UK Public Interest Disclosure Act 1998 (PIDA). It is important to note that the definition of whistleblowing, according to UK law, includes reporting of a criminal offence or reporting information when someone's health and safety is in danger, but does not include personal grievances such as bullying, harassment and discrimination, unless the case is in the public interest [48]. Specifically, whistleblowing on social media is defined as a ‘wide disclosure’ as opposed to disclosures made internally, and is subject to more stringent tests to qualify for protection under the PIDA [49]. Moreover, protection for whistleblowers has been shown to be difficult to enforce, in particular, the law demands an ‘evidential link’ to link the whistleblowing to being fired, which can be difficult to prove [50].

## Implications for the Future

The regulation of online content and communications is a fast‐changing and controversial topic. Many countries are debating how best to legislate in this rapidly evolving component of our societies. For example, the United Kingdom recently implemented the Online Safety Act 2023, which includes a section stating that giving *‘information that the person knows to be false’* and that would risk causing *‘non‐trivial psychological harm’* is a criminal offence [51].

It can be argued that clear and comprehensive regulation of online content and communications under the remit of the law, and by professional regulatory bodies relevant to urologists, will improve protection of the public and trust in the medical profession. However, there has been backlash and criticism of the approach taken by regulators regarding social media use. The UK General Medical Council has stated it *‘has no interest in doctors’ use of social media in their personal lives'* [18]. Yet, their remit covers anything which may be deemed to undermine public trust in the profession. The direction and emphasis of the dual influences of the law and the professional regulators will become clearer as we see more test cases.

The vast majority of urologists will never encounter any issue with their private and public social media use. However, remaining vigilant will reduce the risk of any future pitfalls and perils.

## Summary

As a basic principle, remember that anything said in a text message or email or that is posted online can be repeated back in a court of law or in a professional regulatory hearing. Always consider, before hitting '*send*', whether the content could, in any way, be a source of embarrassment. Remember too, that one's personal communications are no more private than one's professional communications and can be used by the regulator as evidence of impairment to practise. For example, there is no place for racism, sexism or homophobia in medicine. Private social media posts expressing such views risk threatening a doctor's ability to practise. Beyond the regulatory bodies, there is the court of public opinion and, for this, we, as medical professionals, should equally employ the ‘red‐face’ or ‘Daily Mail’ test for social media communications. Protecting our online profile and professional reputation is a career‐long endeavour.

## Disclosure of Interests

H.W.C. and J.R. have no interests to declare.
